# A Psychosocial Intervention for Supporting Informal Caregivers of Older People With Alzheimer Disease: Protocol for the InnFamiglia Randomized Controlled Trial

**DOI:** 10.2196/37496

**Published:** 2022-11-11

**Authors:** Sara Pasquini, Arianna Margaritini, Elena Gambella, Mirko Di Rosa, Elvira Maranesi, Roberta Bevilacqua, Patrizia Civerchia, Giuseppe Pelliccioni

**Affiliations:** 1 IRCCS INRCA Ancona Italy

**Keywords:** Alzheimer disease, caregiver burden, psychosocial intervention, self-help, emotional support, randomized controlled trial, dementia

## Abstract

**Background:**

Dementia is a neurodegenerative syndrome characterized by impaired cognitive functions associated with psychological and behavioral disorders. The informal caregiver has a central role in the life of the person with dementia. Committing a large part of the day to caring for the assisted person inevitably has an effect on the caregiver’s life.

**Objective:**

The aim of this study is to analyze the impact of a psychosocial intervention dedicated to a group of informal caregivers of patients with Alzheimer disease. The intervention will be guided by a trained psychologist who will facilitate the participants’ expression of their emotional states, as compared to a traditional self-help group.

**Methods:**

The intervention described in this paper was designed and developed for the project INNovazione sociale e tecnologica per le FAMIGLIE che assistono malati affetti da Alzheimer (InnFamiglia). The study is designed as a randomized controlled trial (RCT). The RCT includes an experimental group, in which the participants will undertake the psychosocial intervention, and a control group, where participants will receive support according to traditional self-help methodology. Interventions for both groups will last 4 months and will be comprised of 16 sessions.

**Results:**

Participant recruitment, enrollment, and data collection began in 2021. Enrollment continued until September 2022, at which time the last group began the intervention. Data collection will be completed by December 2022, and data analysis will be completed by March 2023. The study findings will be published in peer-reviewed scientific journals and will be presented at scientific meetings. Summaries of the results will also be made available to investigators for dissemination within their clinics.

**Conclusions:**

We hypothesize that the experimental group will be more effective in managing caregiver burden and coping strategies and that this will improve the perception of well-being, anxiety, and depression among caregivers. Our study aims to compare two groups receiving different interventions: a self-help group and a psychosocial group with elements of emotional support. This study may also give us more information about the most appropriate ways to support and help caregivers of people with dementia.

**International Registered Report Identifier (IRRID):**

DERR1-10.2196/37496

## Introduction

Dementia is a neurodegenerative syndrome characterized by impaired cognitive functions associated with psychological and behavioral disorders. Dementia causes a significant reduction in the autonomy of daily life, making it one of the main causes of a lack of self-sufficiency among the older adult population. It is not a specific disease but rather a “family of diseases,” of which Alzheimer disease is the best known and the most prevalent [1].

According to the Dementia Observatory of the Istituto Superiore della Sanità in Italy, the total number of people with dementia is estimated at over 1 million; about 60% of these people have Alzheimer disease. In addition, an estimated 3 million people are directly or indirectly involved in the informal care of people with dementia [2].

Among the European countries, Italy has one of the highest incidences of dementia among its population. In particular, according to the estimates provided by the United Nation’s World Population Prospects from 2018, Italy is second in terms of the number of people with dementia (n=1,279,366); Germany is first, with about 1.5 million people with dementia (n=1,585,166). France follows with an estimated number of people with dementia similar to that of Italy (n=1,227,558). There were estimated to be over 55 million people worldwide living with dementia in 2020. This number will almost double every 20 years, reaching 78 million in 2030 and 139 million in 2050. Much of the increase will be in developing countries. Currently, 60% of people with dementia live in low- and middle-income countries, but by 2050 this number will increase to 71%. The fastest growing older populations live in China and India and in their South Asian and Western Pacific neighboring countries. According to a projection based on these data, in 2050, 4.13% of Italy’s population will be suffering from dementia. This significant increase seems to be correlated with the average rate of aging among the Italian population, with the population older than 85 years doubling [3-5].

Informal caregivers have a central role in the lives of people with dementia because they represent both the person responsible for care and the figure who consistently provides emotional support, day after day.

In most cases, the children of people with dementia play the role of caregiver, particularly daughters; 64.2% of caregivers are the patients’ children. However, the percentage of partners as caregivers is growing—from 25.2% in 2006 to 37% in 2015—especially if the patient is male. This finding also explains the increase in the proportion of sick people living in their own homes, especially if they live alone with their partner [6].

The caregiver devotes an average of 4.4 hours of direct care and 10.8 hours of supervision to the patient with Alzheimer disease each day. Committing a large part of the day to caring for the assisted person inevitably affects the caregiver’s work life. About 59.1% of currently employed caregivers report changes in work life, and the most-cited consequence is repeated absences (37.2%), especially among men (62.5%). Women, compared to men, indicate more frequently that they have requested part-time work schedules (26.9%). In addition, 29.5% of caregivers take time off from work [6]. Among currently unemployed caregivers, 18.7% reported changes in work life that in some cases coincided with the most extreme consequence: job loss [6].

The caregiver’s commitment also has consequences on their state of health, especially among women, and shows up as fatigue, reported by 80.3% of women as compared to 68.8% of men; lack of sleep (63.2%); symptoms of depression (45.3%); and frequent diseases (26.1%). In addition, a large percentage of caregivers take medications due to the health consequences caused by the immense commitment involved in the care of the patient [6]. There are also negative effects on the caregiver’s relational and social life, from the interruption of activities outside of work (76.0%) to the negative impact on family members (59.7%) and friendships (45.6%) [6].

Caregiver burden is a syndrome that represents the degree to which caregivers negatively perceive their own stress as a result of taking care of their assisted family member. The caregiver can experience problems from a multidimensional perspective, at emotional, social, financial, physical, and spiritual levels [7].

Risk factors for caregiver burden include the following: female sex, low level of education, living with the cared-for person, depression, social isolation, financial strain, high number of hours caring for the sick person, and no other help in caring [8].

Caregivers may suffer from various diseases, such as depression; may have inadequate coping strategies; and may perceive themselves as having a poor quality of life. This may result in physical and psychological symptoms, with which they try to cope by abusing drugs, as compared to noncaregivers [1,9,10]. Support for caregivers is also essential for the well-being of people with dementia. In fact, caregiver characteristics and their perceived burden are factors that are related to the well-being of the person with dementia: high caregiver burden is associated with a higher use of antipsychotic drugs by the patient and a higher risk of institutionalization [11].

Meta-analysis studies revealed that psychosocial interventions had significant effects on caregiver burden, depression, and general health, although the overall effect on quality of life was not statistically significant. The literature also suggests that psychoeducational programs may offer the greatest benefit in relation to reducing the burden of care, while multicomponent and alternative interventions may be better suited to coping with depression [12].

Even cognitive behavioral approaches are promising, especially for the reduction of depressive symptoms, while psychoeducational interventions can instead reduce the subjective load of the caregiver. In particular, the subjective load seems to be the factor that most improves with psychosocial interventions for the caregiver [13]. As seen in the literature, the approaches used to support caregivers focus more on educational support, education, social skills, cognitive behavioral support, information, and psychoeducational support [14].

Among the interventions for caregivers, support groups have been shown to be effective. In this type of group, caregivers meet each other and freely discuss their practical experiences and their emotional experiences, in particular. These groups can help the person experience the difficult task of caregiving more calmly and consciously.

Caregivers may have the opportunity to discuss common problems, to receive useful information from other people living in similar situations, to increase their coping strategies, and to have emotional support regarding the experience of the disease [6,15,16].

Support groups are one of the most important interventions: people face the same problems and share their experiences through mutual help [15,17-19]. These kinds of groups provide the caregiver with a comfortable environment where they can share their emotions, reduce stress and caregiver burden, alleviate social isolation, and improve quality of life. The World Alzheimer Report 2015 reported that there is a need to strengthen services for caregivers of Alzheimer patients in order to reduce their burden by making health workers aware that caregivers are an essential part of ongoing care for the Alzheimer patient [20,21].

Following these reviews in the literature, it seems important to assess the effectiveness of group interventions in supporting the family caregiver of the person with Alzheimer disease.

The intervention described in this paper was designed and developed for the project INNovazione sociale e tecnologica per le FAMIGLIE che assistono malati affetti da Alzheimer (InnFamiglia), funded by Fondazione Cariverona. The project aimed to support older people affected by Alzheimer disease and their families throughout the duration of the disease, from initial subjective memory complaints to the severe dementia phase, overcoming the fragmentation of the services available in the local community. The project, in fact, will exploit an ecosystem of innovative services, designed in a participatory approach with the older patients, their families, and local stakeholders. The services will include the following: the development of physical and cognitive interventions, integrated with technological solutions to slow down and prevent disease progression; the availability of a daily care center for patients at the mild to moderate stages; the delivery of specialized training to a multidisciplinary team for home assistance of patients at the severe stage; and the provision of tailored interventions to support and educate informal caregivers, such as the one discussed in this paper. The study presented in this paper aims to analyze the impact of a psychosocial intervention provided to a group of informal caregivers of patients with Alzheimer disease, guided by a trained psychologist for facilitating the expression of emotional states, compared to a traditional self-help group. The factors observed and evaluated in the assessment will be the caregivers’ depression and coping strategies, caregiver burden, and their perceived quality of life.

## Methods

### Study Design

**Figure 1:**
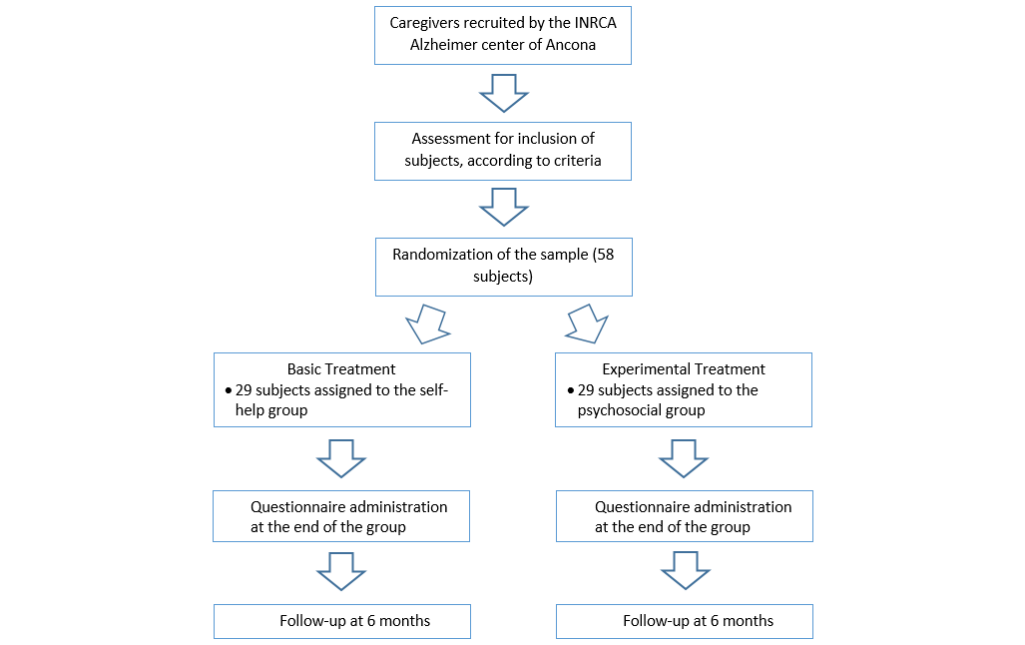
Experimental design of the randomized controlled trial. INRCA: Istituto Nazionale di Ricovero e Cura per Anziani.

The study is designed as a randomized controlled trial (RCT). The RCT will include an experimental group, in which participants will undertake the psychosocial intervention, and a control group, where participants will receive support according to the traditional self-help methodology. Interventions for both groups will last 4 months and will be comprised of 16 sessions. The following evaluation phases are planned: T0 (baseline), T1 (end of treatment), and T2 (follow-up at 6 months). At each stage, a self-assessment questionnaire will be administered as described in Multimedia Appendix 1. Figure 1 describes the experimental design.

### Participants, Recruitment, and Sample Size

The sample consists of family caregivers of people with Alzheimer disease who are followed by the Alzheimer Center of the Istituto di Ricovero e Cura a Carattere Scientifico (IRCCS) Istituto Nazionale di Ricovero e Cura per Anziani (INRCA) of Ancona. No limits are set for the ages of both the patient and the caregiver, nor for the type of family relationship between them.

Eligibility criteria for caregivers include the following:

- Signed an informed consent form
- Aged 18 years or older
- Is a caregiver for a person with mild to moderate Alzheimer disease
- The person with Alzheimer disease resides at their home or at the patient’s own home.

Exclusion criteria include the following:

- The caregiver failed to meet the inclusion criteria
- The family member with Alzheimer disease has psychiatric disorders that were detected before the diagnosis of Alzheimer disease
- The family members of the patient with Alzheimer disease have psychiatric disorders that were detected before the diagnosis of Alzheimer disease.

The type of intervention hypothesized for this study and the expected efficacy for the case group is comparable to the work of Küçükgüçlü et al [15]. The authors of this work, which included 30 individuals, obtained an average reduction in caregiver burden of 4.61 points, corresponding to an effect size of 0.243.

Assuming the same effect size in our group of cases, setting the statistical power at 90% and a significance level of .05, it was estimated through repeated-measures within-factors analysis of variance (ANOVA)—two groups and two measurement times (ie, baseline and follow-up)—that the total sample size expected must be at least 48 subjects. Considering a dropout rate of about 20%, the sample size increased to 58 subjects: 29 cases and 29 controls.

Recruitment was carried out during daily visits to the Alzheimer Centre of the IRRCS INRCA in Ancona, where the older person was accompanied by a family member. Once the criteria for participation were established and met, a proposal to join a support group was made, the caregiver chose whether or not to participate, and the caregiver was randomly placed in one of the two groups.

### Randomization

This study has a single-blind design, as far as the evaluator is concerned, since the characteristics of the treatment do not allow for double- or triple-blind experimental procedures. A randomization technique based on a single sequence of random assignments was used. A list of random numbers generated by the computer was used, and subjects were assigned a number based on their order of inclusion in the study. According to the technique described above, the 58 subjects were randomly assigned to one of the two study groups. Randomized subjects leaving the study will not be replaced, as a dropout percentage has already been considered when estimating the sample size.

### Intervention

In this study, we will facilitate two different types of support groups for a sample of caregivers.

Regarding the experimental group, the minimum number of participants will be 3 and the maximum number will be 12. This group includes 16 meetings: 1 to present the study to the participants and for administration of the questionnaire; 14 dedicated to discussions led by the facilitator, who presents topics, as described below; and a final meeting dedicated to the administration of the questionnaire.

The protocol of the psychosocial intervention includes the following topics:

- First meeting—“Presentation of the participants of the group and administration of the questionnaire.” The facilitator will present the activity, the participants will introduce themselves, and the questionnaire will be administered.
- Second and third meetings—“Let’s get to know Alzheimer disease: what is it? Disease and epidemiology.” The facilitator will explain what Alzheimer disease is, providing theoretical information about the disease and its epidemiology. This allows caregivers to get a first complete picture of their family member’s disease.
- Fourth and fifth meetings—“Let’s get to know Alzheimer disease: what is it? Symptoms and phases.” The facilitator will give further theoretical information on Alzheimer disease, focusing in particular on the stages of the disease, descriptions of the stages, and the symptoms of the disease.
- Sixth and seventh meetings—“Our changing family member: how to relate to him/her?” The facilitator will introduce the topic concerning the changes of the ill family member due to the symptoms of dementia. In particular, the facilitator will try to focus on relational changes between the caregiver and family member, providing useful information on how to relate.
- Eighth and ninth meetings—“Parents and children, husband and wife: the changing family.” The facilitator will introduce the topic of changes in the family structure as a result of the Alzheimer disease event, expose what are the most common changes, and describe strategies for dealing with them.
- Tenth and eleventh meetings—“Being a caregiver: the changes in our lives.” The facilitator will introduce the topic of personal changes in the caregiver’s life and what can be useful to improve his or her condition. The facilitator will expose the most common changes in the caregiver’s life, focusing on the importance for the caregiver to take care of himself or herself.
- Twelfth and thirteenth meeting—“Let’s get to know each other better: emotions and photography.” Each participant will bring personal photos that represent his or her life as a caregiver. Through the photos, each participant will talk about his or her own caregiving experience and story.
- Fourteenth and fifteenth meetings—“The emotions and experiences of being a caregiver.” The facilitator will give space to the participants’ emotions centered on being a caregiver.
- Sixteenth meeting—Administration of the questionnaire and conclusion of the group intervention. The facilitator will collect thoughts and reflections at the end of the group and will administer the questionnaire.

Regarding the control group, the minimum number of participants will be 3 and the maximum number will be 12. This group includes 16 meetings: 1 for the presentation of the study to the participants and for the administration of the questionnaire, 14 for free discussions, and 1 final meeting for the administration of the questionnaire and conclusion of the group intervention. The control group will follow self-help methodology in such a way that the facilitator will have the sole task of facilitating communication between members of the group as the topics are chosen and treated freely by participants.

For both groups, each session will last 1.5 hours. During the first meeting for both groups, the informed consent forms with explanations about the intervention will be completed and the first questionnaires will be completed. In the final meeting, participants from both groups will complete the final questionnaire; after 6 months, they will be contacted by the researchers for the follow-up assessment.

### Outcome Measures

#### Primary Outcome

The primary outcome is caregiver burden, which will be evaluated using the Caregiver Burden Inventory scale [22]. This tool evaluates the care load, analyzing its multidimensional aspect, and was developed for caregivers of patients with Alzheimer disease and related dementias. It consists of 24 questions that are grouped into five sections representing stress-related factors: objective load (questions 1-5), evolutionary load (questions 6-10), physical load (questions 11-14), social load (questions 15-19), and emotional load (questions 20-24). Each statement is assigned a value on a 5-degree scale of increasing activity.

#### Secondary Outcomes

Secondary outcomes include coping strategy, measured using the Coping Orientation to Problems Experienced (COPE) instrument; depression, measured using the Hospital Anxiety and Depression Scale (HADS); and quality of life, measured using an ad hoc constructed questionnaire.

The COPE instrument is a self-report questionnaire that considers different coping modalities. The questionnaire consists of 60 items. The questionnaire evaluates how often the subject uses particular coping processes in difficult or stressful situations. There are four possible responses, ranging from “I usually don’t do it” to “I almost always do it.” The 15 different coping mechanisms considered by the questionnaire are as follows: activity, planning, suppression of competitive activities, containment, seeking information, seeking understanding, emotional venting, positive reinterpretation and growth, acceptance, devotion to religion, humor, denial, behavioral detachment, mental detachment, and drug or alcohol use [23].

The HADS is an instrument that measures symptoms of anxiety and depression and consists of 14 items: seven for the anxiety subscale and seven for the depression subscale. With regard to anxiety, we will focus mainly on the symptoms of generalized anxiety disorder; regarding depression, we will focus on anhedonia, the main symptom of depression. Scores range from 0 to 3 for each item. For each of the statements, the subject is asked which of four possible options best describes their emotional state [24].

The perception of quality of life will be assessed through four ad hoc items that investigate the person’s experience with respect to the quality of his or her leisure time, health status, mood, and social relationships. Each item has five possible responses, with scores ranging from 0 to 5.

In addition, a complete checklist on the demographic characteristics of the sample will be collected, with the aim of profiling the participants on the basis of their living arrangements, income, past occupational activity, family composition, age, and gender. Those background variables will be used in descriptive statistics to evaluate their influence on the study outcomes.

### Data Analysis

A descriptive analysis of the sample will be carried out through univariate and bivariate statistical analysis. Continuous variables will reported as mean and SD or median and IQR, based on their distribution, and will be evaluated using the Shapiro-Wilk test. The comparison of variables between groups at baseline will be carried out by means of unpaired Student *t* tests or Mann-Whitney *U* tests, based on their distribution. Categorical variables will be expressed as absolute frequencies and percentages, and statistical significance will be evaluated using chi-square tests or Fisher exact tests, in the case of comparisons between small subsamples.

In the second phase, the analysis of follow-up data will be carried out in order to assess the effectiveness of the intervention. This phase of analysis involves the use of multivariate statistical techniques, in particular, repeated-measures ANOVA, in order to compare changes in outcome measures over time between the intervention group and the control group. In the case of different exposure times between subjects, the Cox regression model will be employed in order to identify factors associated with the change in key endpoints. Statistical significance will be set at *P*<.05.

Every effort will be made to collect all data within the specified time frame. In the case of missing and unrecoverable data on the primary endpoints, we will assume that these events are related to chance. Analyses will be performed by applying list-wise deletion to remove cases with missing values from the final database in order to obtain unbiased estimates.

### Ethics Approval

The study was approved by the Ethics Committee of the IRCCS INRCA on November 26, 2020 (approval No. CE INRCA 20018). The Ethics Committee will be notified about any protocol modifications. The Ethics Committee is in charge of data monitoring and will periodically assess the progress of the protocol and compliance with what was declared. The study will adhere to the principles of the Declaration of Helsinki and Good Clinical Practice guidelines. Participants in this study will provide written informed consent.

## Results

Participant recruitment, enrollment, and data collection began in 2021. Enrollment continued until September 2022, at which time the last group began the intervention. Data collection will be completed by December 2022, and data analysis will be completed by March 2023. The study findings will be published in peer-reviewed scientific journals and will be presented at scientific meetings. Summaries of the results will also be made available to investigators for dissemination within their clinics.

## Discussion

Despite several available studies, there is still moderate evidence on the efficacy of psychosocial interventions for dementia caregivers. In fact, although understanding the needs of caregivers is essential for developing effective interventions, only a few systematically address them.

With this study, we expect to advance the state of the art in the field by evaluating the efficacy of a self-help intervention using an evidence-based approach. We will do this through an RCT study, aimed at improving caregivers’ quality of life, reducing their care burden, and easing their anxiety or depressive symptoms, which represent the most felt needs of the population of carers. Moreover, the study will also identify barriers and obstacles that may arise during the organization of group interventions, especially in the COVID-19 era, in which isolation and restriction in mobility may affect participation and relationship-building among the group.

The aim of this study is to analyze the impact of a psychosocial intervention dedicated to a group of informal caregivers of patients with Alzheimer disease; the intervention will be guided by a trained psychologist in order to facilitate the expression of the group members’ emotional states and will be compared to a traditional self-help group.

We focused on patients with Alzheimer disease because dementia is becoming one of the main conditions, worldwide, that causes a lack in self-sufficiency [25]. It is estimated that about 3 million people are directly or indirectly involved in the care and support of people with dementia. In particular, informal caregivers (ie, family members) [26,27] play a fundamental role in the lives of people with dementia because they provide both physical and psychological support to ill family members on a daily basis [28]. Therefore, it is important to provide support to the caregiver [29]. Our study compares two groups receiving different interventions: a self-help group and a psychosocial group with elements of emotional support.

The self-help group, based on its typical characteristics, is not a structured group and leaves the content to be decided by the participants. The experimental group is structured around topics defined by the facilitator or psychologist, focused on the experiences and needs of the caregivers, with particular attention paid to their personal emotional experience. We hypothesize that the experimental group will be more effective in managing caregiver burden and coping strategies, which could improve the perception of well-being, anxiety, and depression among caregivers. This is because since the experimental group is more structured, it provides more skills, advice, and support than the self-help group. This study may also give us more information about the most appropriate ways to support and help caregivers of people with dementia.

## Appendix

Multimedia Appendix 1 Self-assessment questionnaire (in Italian).

## Abbreviations

ANOVA: analysis of variance
COPE: Coping Orientation to Problems Experienced
HADS: Hospital Anxiety and Depression Scale
InnFamiglia: INNovazione sociale e tecnologica per le FAMIGLIE che assistono malati affetti da Alzheimer
INRCA: Istituto Nazionale di Ricovero e Cura per Anziani
IRCCS: Istituto di Ricovero e Cura a Carattere Scientifico
RCT: randomized controlled trial
