# Supplementary material for: A Psychosocial Intervention for Supporting Informal Caregivers of Older People With Alzheimer Disease: Protocol for the InnFamiglia Randomized Controlled Trial
Source: JMIR Res Protoc. 2022 Nov 11;11(11):e37496. doi: 10.2196/37496 (PMC9700238; doi:10.2196/37496)
Supplement: Multimedia Appendix 1 [file resprot_v11i11e37496_app1.docx]

QUESTIONARIO SOMMINISTRATO AI CAREGIVER

**Le chiediamo di rispondere ad alcune domande *che riguardano il suo familiare malato***

Sesso

- Maschio
- Femmina

Età ………………..

Stato civile

- Celibe-Nubile
- Coniugato/a
- Separato/a
- Divorziato/a
- Vedovo/a

Scolarità

- Analfabeta
- Nessun titolo di studio ma sa leggere e scrivere
- Licenza elementare
- Licenza scuola media
- Diploma scuola superiore
- Laurea
- Altro (specioficare)…………………

Anno esordio della malattia …………..

Il suo familiare ha una certificazione di invalidità civile?

- Si
- No
- In attesa di richiesta

Se si in che percentuale? …………………%

Il suo familiare è in possesso di una indennità di accompagno?

- Si
- Non concessa
- Non richiesta
- In attesa di richiesta

Il suo familiare frequenta o usufruisce di uno di questi servizi:

- Centro diurno Alzheimer
- Centro sociale
- Centro Disturbi Cognitivi e Demenze
- Assistenza domiciliare riabilitativa
- Assistenza domiciliare programmata
- Assistenza domiciliare integrata
- Assistenza infermieristica programmata
- Pasti a domicilio
- Altro (specificare) ………………………….

Il suo familiare usufruisce di badanti o altre persone a pagamento?

- Si
- No

**Se sì**, da quanto tempo? ...............................................

Per quante ore al giorno? .................... Per quanti giorni alla settimana ....................

Il suo familiare è in terapia farmacologica?

- Si
- No

**Se Sì**, usufruisce di un piano terapeutico?

- Si
- No

**Se Sì**, ricorda i farmaci prescritti?

…………………………………………………………………………………………….

Attualmente con chi vive il suo familiare?

- Da solo
- Con il coniuge/ compagno/a
- Figli
- Coniuge e figli
- Fratelli/sorelle
- Altri parenti
- Assistenti a pagamento (badanti ecc..)
- Altro (specificare) ……………………….

Il suo familiare è:

- Lavoratore/lavoratrice
- Pensionato/a
- Disoccupato/a

**Le chiediamo ora di rispondere ad alcune domande *che la riguardano direttamente.***

Sesso

- Maschio
- Femmina

Età ……………….

Grado parentela con il familiare malato

- Coniuge
- Convivente
- Figlio/a
- Nuora/Genero
- Fratello/sorella
- Altro ………………….

Convive con il familiare malato?

- Si
- No

Titolo di studio

- Licenza elementare
- Licenza media
- Diploma scuola superiore
- Laurea
- Altro………

Qual è la sua situazione lavorativa?

- Lavoratore/lavoratrice dipendente
- Lavoratore/lavoratrice autonomo
- Pensionato/a
- Disoccupato/a
- Altro (specificare)…………………………….

Tipo di professione…………………………………..

Ha cessato il lavoro a causa dell’assistenza al familiare malato?

- Si
- No

Ha diminuito il suo orario lavorativo a causa dell’assistenza al familiare malato?

- Si
- No

Quante ore al giorno, in media, deve dedicare a supervisionare il familiare malato? (Ad es. verificare l’assunzione dei farmaci, dei pasti e dell’igiene personale ecc.)

- O ore
- Circa 2-4 ore
- Circa 5-7 ore
- Circa 8 ore
- Più di 8 ore

Quante ore al giorno, in media, deve dedicare per assistere direttamente il familiare malato? (Ad es. fargli il bagno, preparare i vestiti, preparare il pranzo ecc.)

- 0 ore
- Circa 2-4 ore
- Circa 5-7 ore
- Circa 8 ore
- Più di 8 ore

Ha familiari o amici che collaborano con lei?

- Si
- No

Se si quanti?

- 1
- 2
- 3
- Più di tre

**Ora si concentri su se stesso e risponda, per favore, a queste domande…**

Come descriverebbe, complessivamente il **suo** stato di salute attuale?

- Pessimo
- Mediocre
- Discreto
- Buono
- Ottimo

Come descriverebbe complessivamente il **suo** umore ed il suo stato d’animo?

- Pessimo
- Mediocre
- Discreto
- Buono
- Ottimo

Come descriverebbe complessivamente la qualità delle **sue** relazioni sociali e la sua vita sociale?

- Pessima
- Mediocre
- Discreta
- Buona
- Ottima

Come descriverebbe complessivamente la qualità del **suo** tempo libero e di svago/relax?

- Pessima
- Mediocre
- Discreta
- Buona
- Ottima

Ora risponda, per favore, a queste domande scegliendo la risposta che più corrisponde a come lei si è sentito **nell’ultima settimana** compreso oggi.

1. Mi sento agitato e teso:

- Quasi sempre
- Molto spesso
- Qualche volta
- Mai

1. Le cose che un tempo mi piacevano, ora mi piacciono:

- Nello stesso modo
- Meno di prima
- Molto poco
- Quasi per niente

1. Provo un sentimento di apprensione, come se dovesse succedere qualcosa di terribile:

- Sicuramente e in modo intenso
- Si, ma non troppo intenso
- Un po', ma non da preoccuparmene
- Per niente

1. Mi passano per la mente pensieri preoccupanti:

- Molto spesso
- Spesso
- A volte, non troppo spesso
- Solo ogni tanto

1. Mi passano per la mente pensieri preoccupanti:

- Molto spesso
- Spesso
- A volte, non troppo spesso
- Solo ogni tanto

1. Mi sento di buon umore:

- Mai
- Raramente
- Qualche volta
- Quasi sempre

1. Riesco a sedermi e rilassarmi:

- Sempre
- Spesso
- Qualche volta
- Mai

1. Mi sento rallentato:

- Quasi sempre
- Molto spesso
- Qualche volta
- Mai

1. Provo un sentimento simile alla paura, come con un senso di tensione allo stomaco:

- Mai
- Qualche volta
- Abbastanza spesso
- Molto spesso

1. Non curo più il mio aspetto fisico:

- Completamente
- Non me curo abbastanza
- Forse me ne curo di meno
- Me ne curo come al solito

1. Mi sento irrequieto e incapace di stare fermo

- Moltissimo
- Abbastanza
- Poco
- Per niente

1. Pregusto con piacere le cose

- Come ho sempre fatto
- Un po' meno di un tempo
- Molto meno di un tempo
- Non vi riesco affatto

1. Provo delle sensazioni improvvise di panico:

- Molto spesso
- Abbastanza spesso
- Non molto spesso
- Molto raramente

1. Provo piacere leggendo un libro, oppure seguendo una trasmissione alla radio o alla TV:

- Spesso
- Qualche volta
- Non spesso
- Molto raramente

Per favore cerchi di rispondere a una domanda per volta e non tenga conto delle risposte che ha dato precedentemente. Scelga con attenzione la risposta che meglio descrive il suo modo di comportarsi. Per favore risponda a ogni affermazione. Non ci sono risposte giuste o sbagliate, perciò scelga la risposta più appropriata per lei e non quella che la maggior parte delle persone, secondo lei, darebbe.

**Risponda a ciascuna delle seguenti affermazioni segnando il numero che corrisponde alla sua risposta.**

I numeri vanno interpretati in questo modo:

**1 = Di solito non lo faccio.**

**2 = Lo faccio qualche volta.**

**3 = Lo faccio con una certa frequenza.**

**4 = Lo faccio quasi sempre.**

| 1. Cerco di utilizzare questa esperienza per crescere come persona. 1 2 3 4 |
| --- |
| 2. Mi dedico al lavoro o ad altre attività per non pensare a ciò che mi preoccupa. 1 2 3 4 |
| 3. Mi preoccupo e do sfogo alle mie emozioni. 1 2 3 4 |
| 4. Cerco di farmi consigliare da qualcuno sul da farsi. 1 2 3 4 |
| 5. Mi impegno al massimo per agire sulla situazione. 1 2 3 4 |
| 6. Dico a me stesso/a che sto vivendo qualcosa di irreale. 1 2 3 4 |
| 7. Ripongo la mia speranza in Dio. 1 2 3 4 |
| 8. Rido della situazione. 1 2 3 4 |
| 9. Riconosco che non posso farci niente e abbandono ogni tentativo di agire. 1 2 3 4 |
| 10. Mi trattengo dall’agire troppo in fretta. 1 2 3 4 |
| 11. Parlo dei miei sentimenti con qualcuno. 1 2 3 4 |
| 12. Faccio uso di alcool o droghe o farmaci per sentirmi meglio. 1 2 3 4 |
| 13. Cerco di abituarmi all’idea che ciò è successo. 1 2 3 4 |
| 14. Parlo con qualcuno per capire di più sulla situazione. 1 2 3 4 |
| 15. Non mi faccio distrarre da altri pensieri o attività. 1 2 3 4 |
| 16. Tendo a fantasticare per distrarmi. 1 2 3 4 |
| 17. Mi rendo conto di preoccuparmi molto. 1 2 3 4 |
| 18. Cerco aiuto in Dio. 1 2 3 4 |
| 19. Preparo un piano d’azione. 1 2 3 4 |
| 20. Ci scherzo sopra. 1 2 3 4 |
| 21. Accetto che ciò sia accaduto e che non possa essere cambiato. 1 2 3 4 |
| 22. Mi trattengo dal fare qualsiasi cosa fi no a che la situazione lo permetta. 1 2 3 4 |
| 23. Cerco sostegno morale dagli amici e dai parenti. 1 2 3 4 |
| 24. Semplicemente rinuncio a raggiungere i miei obiettivi. 1 2 3 4 |
| 25. Mi sforzo più del solito per tentare di liberarmi dal problema. 1 2 3 4 |
| 26. Cerco di distrarmi per un momento bevendo alcool o prendendo droghe o farmaci. 1 2 3 4 |
| 27. Mi rifiuto di credere che ciò sia accaduto. 1 2 3 4 |
| 28. Do libero sfogo ai miei sentimenti. 1 2 3 4 |
| 29. Cerco di vedere le cose in una luce diversa, per farle sembrare più positive. 1 2 3 4 |
| 30. Parlo con qualcuno per fare qualcosa di concreto per risolvere il problema. 1 2 3 4 |
| 31. Dormo più del solito. 1 2 3 4 |
| 32. Cerco di escogitare una strategia sul da farsi. 1 2 3 4 |
| 33. Mi concentro nel trattare questo problema, e se necessario |
| metto da parte le altre cose. 1 2 3 4 |
| 34. Cerco la comprensione e la solidarietà di qualcuno. 1 2 3 4 |
| 35. Bevo alcool o prendo droghe o farmaci per pensarci di meno. 1 2 3 4 |
| 36. Mi prendo gioco di quello che è successo. 1 2 3 4 |
| 37. Rinuncio a ogni tentativo di ottenere ciò che voglio. 1 2 3 4 |
| 38. Cerco qualcosa di positivo in ciò che sta accadendo. 1 2 3 4 |
| 39. Penso a come potrei gestire al meglio il problema. 1 2 3 4 |
| 40. Faccio finta che non sia veramente accaduto. 1 2 3 4 |
| 41. Mi accerto di non peggiorare le cose agendo troppo presto. 1 2 3 4 |
| 42. Cerco strenuamente di impedire che le altre cose interferiscano |
| con i miei sforzi di fronteggiare il problema. 1 2 3 4 |
| 43. Vado al cinema o guardo la televisione per pensarci di meno. 1 2 3 4 |
| 44. Accetto la realtà dei fatti. 1 2 3 4 |
| 45. Chiedo alle persone come hanno agito di fronte ad esperienze simili. 1 2 3 4 |
| 46. Mi sento molto stressato/a e lascio andare i miei sentimenti. 1 2 3 4 |
| 47. Agisco senza indugio per sbarazzarmi del problema. 1 2 3 4 |
| 48. Tento di trovare conforto nella mia religione. 1 2 3 4 |
| 49. Mi sforzo di aspettare il momento giusto per fare qualcosa. 1 2 3 4 |
| 50. Tento di ridicolizzare la situazione. 1 2 3 4 |
| 51. Non mi impegno più di tanto per risolvere il problema. 1 2 3 4 |
| 52. Parlo con qualcuno di come mi sento. 1 2 3 4 |
| 53. Faccio uso di alcool o droghe o farmaci per aiutarmi ad uscirne. 1 2 3 4 |
| 54. Imparo a convivere con il problema. 1 2 3 4 |
| 55. Metto da parte le altre attività per concentrarmi sulla situazione. 1 2 3 4 |
| 56. Rifl etto intensamente su quali mosse fare. 1 2 3 4 |
| 57. Mi comporto come se non fosse mai accaduto. 1 2 3 4 |
| 58. Faccio quello che deve essere fatto, un passo alla volta. 1 2 3 4 |
| 59. Tento di imparare qualcosa dall’esperienza. 1 2 3 4 |
| 60. Prego più del solito. 1 2 3 4 |
|  |

Le domande si riferiscono a lei che assiste il suo congiunto malato; risponda mettendo una crocetta sopra il numero che più si avvicina alla sua condizione o alla sua personale impressione seguendo la legenda.

# LEGENDA 0 Per nulla

**1 Un poco**

**2 Moderatamente**

**3 Parecchio**

**4 Molto**

| **1.** Il mio familiare necessita del mio aiuto per svolgere molte delle abituali attività quotidiane. | 0 1 2 3 4 |
| --- | --- |
| **2.** Il mio familiare è dipendente da me. | 0 1 2 3 4 |
| **3.** Devo vigilarlo costantemente. | 0 1 2 3 4 |
| **4.** Devo assisterlo anche per molte delle più semplici attività quotidiane (vestirlo, lavarlo, uso dei servizi igienici). | 0 1 2 3 4 |
| **5.** Non riesco ad avere un minuto di libertà dai miei compiti di assistenza. | 0 1 2 3 4 |
| **6.** Sento che sto perdendo la mia vita. | 0 1 2 3 4 |
| **7.** Desidero poter fuggire da questa situazione. | 0 1 2 3 4 |
| **8.** La mia vita sociale ne ha risentito. | 0 1 2 3 4 |
| **9.** Mi sento emotivamente svuotato a causa del mio ruolo di assistente. | 0 1 2 3 4 |
| **10.** Mi sarei aspettato qualcosa di diverso a questo punto della mia vita | 0 1 2 3 4 |
| **11.** Non riesco a dormire a sufficienza. | 0 1 2 3 4 |
| **12.** La mia salute ne ha risentito. | 0 1 2 3 4 |
| **13.** Il compito di assisterlo mi ha reso più fragile di salute. | 0 1 2 3 4 |
| **14.** Sono fisicamente stanco. | 0 1 2 3 4 |
| **15.** Non vado d’accordo con gli altri membri della famiglia, come di consueto. | 0 1 2 3 4 |
| **16.** I miei sforzi non sono considerati dagli altri familiari. | 0 1 2 3 4 |
| **17.** Ho avuto problemi con il coniuge | 0 1 2 3 4 |
| **18.** Sul lavoro non rendo come di consueto. | 0 1 2 3 4 |
| **19.** Provo risentimento verso i miei familiari che potrebbero darmi una mano ma non lo  fanno. | 0 1 2 3 4 |
| **20.** Mi sento imbarazzato a causa del comportamento del mio familiare. | 0 1 2 3 4 |
| **21.** Mi vergogno di lui. | 0 1 2 3 4 |
| **22.** Provo risentimento nei suoi confronti. | 0 1 2 3 4 |
| **23.** Non mi sento a mio agio quando ho amici in casa. | 0 1 2 3 4 |
| **24.** Mi arrabbio per le mie reazioni nei suoi riguardi. | 0 1 2 3 4 |

Come valuta complessivamente la qualità di vita del suo familiare malato?

- Pessima
- Mediocre
- Discreta
- Buona
- Ottima

Rispetto a sei mesi fa, la definirebbe:

- Migliore
- Invariata
- Peggiore

Io penso che: (si possono fornire più di una risposta indicando l’ordine di importanza)

- Maggiori conoscenze sulla malattia potrebbero aiutarmi.
- Niente mi può aiutare.
- I servizi dovrebbero risolvere concretamente i problemi legati alla malattia.
- Se riuscissi ad adattarmi alla malattia del mio familiare tutto andrebbe meglio.
- Se avessi a disposizione 24 ore su 24 un numero telefonico dove trovare conforto, aiuto e consiglio psicologico e medico, potrei affrontare meglio la salute del mio familiare.
- Nessuno può offrire il mio stesso tipo di assistenza. Nessuno può sostituirmi.
- I servizi mi aiutano fornendomi indicazioni mediche, psicologiche e sociali utili per affrontare al meglio la malattia del mio familiare.

Che cosa si aspetta dal frequentare un gruppo di auto mutuo aiuto? (E’ possibile scegliere più di una risposta indicando l’ordine di importanza)

- Ottenere informazioni e maggiori conoscenze riguardanti la malattia
- Poter essere di aiuto, attraverso la mia esperienza, a chi si trova nelle mie stesse condizioni
- Essere ascoltato nelle mie difficoltà
- Trovare strategie migliori nella cura e gestione del mio familiare malato
- Trovare strategie migliori nella gestione del mio carico psicologico
- Non mi aspetto nulla
- Trovare persone che mi possono aiutare concretamente nell’assistenza del mio familiare
- Trovare delle persone che mi capiscono
- Trovare un luogo dove poter sfogare i miei problemi
- Altro (specificare) ………………………………………………………………………………………………………

**Grazie per la sua collaborazione**
